# Complex Relationship Between Cardiac Fibroblasts and Cardiomyocytes in Health and Disease

**DOI:** 10.1161/JAHA.120.019338

**Published:** 2021-02-15

**Authors:** Caitlin Hall, Katja Gehmlich, Chris Denning, Davor Pavlovic

**Affiliations:** ^1^ Institute of Cardiovascular Sciences University of Birmingham United Kingdom; ^2^ Division of Cardiovascular Medicine Radcliffe Department of Medicine and British Heart Foundation Centre of Research Excellence Oxford University of Oxford United Kingdom; ^3^ Biodiscovery Institute University of Nottingham United Kingdom

**Keywords:** arrhythmias, cardiac fibroblasts, cardiomyocytes, fibrosis, heart failure, myofibroblast, Arrhythmias, Electrophysiology, Animal Models of Human Disease, Fibrosis

## Abstract

Cardiac fibroblasts are the primary cell type responsible for deposition of extracellular matrix in the heart, providing support to the contracting myocardium and contributing to a myriad of physiological signaling processes. Despite the importance of fibrosis in processes of wound healing, excessive fibroblast proliferation and activation can lead to pathological remodeling, driving heart failure and the onset of arrhythmias. Our understanding of the mechanisms driving the cardiac fibroblast activation and proliferation is expanding, and evidence for their direct and indirect effects on cardiac myocyte function is accumulating. In this review, we focus on the importance of the fibroblast‐to‐myofibroblast transition and the cross talk of cardiac fibroblasts with cardiac myocytes. We also consider the current use of models used to explore these questions.

**Nonstandard Abbreviations and Acronyms**
Ang II: angiotensin II
cFb: cardiac fibroblast
Cx43: connexin 43
ECM: extracellular matrix
FSP1: fibroblast‐specific protein 1
hiPSC: human induced pluripotent stem cell
SMAD: small mothers against decapentaplegic
TGF‐β: transforming growth factor‐β
α‐SMA: α‐smooth muscle actin

Because of aging populations and lifestyle changes, the total number of deaths caused by cardiovascular diseases (CVDs) is increasing, by ≈21% from 2007 to 2017.^1^ High mortality rates as well as a high level of morbidity contribute to the high economic burden of CVDs.^2^ Many of these deadly and debilitating CVDs are associated with cardiac fibrosis, which can be classified as either diffuse interstitial or replacement fibrosis.^3^ Replacement fibrosis occurs immediately following cardiac injury, such as myocardial infarction (MI), to form a fibrotic scar preventing rupture of the myocardium.^3^, ^4^ Interstitial fibrosis is associated with inflammation and more chronic conditions, such as cardiomyopathies or hypertension, and if the underlying condition is left untreated, results in irreversible replacement fibrosis.^3^, ^4^, ^5^ Cardiac fibrosis contributes to both electrical and structural remodeling of the heart, which ultimately leads to decreased cardiac function, heart failure, and arrhythmias.^4^, ^6^, ^7^, ^8^ The relationship between fibrosis and CVDs has been the subject of multiple studies. However, with no efficient treatments directly targeting cardiac fibrosis, it presents an ever‐growing clinical challenge.^9^, ^10^ The fibrotic process is mediated by activation of cardiac fibroblasts (cFbs), an important nonexcitatory cell population in the heart, responsible for deposition of extracellular matrix (ECM) in health and disease. cFbs can influence cardiac function through direct and indirect effects on cardiomyocytes.^11^, ^12^, ^13^ In this review, we summarize the current understanding of the interactions between cFbs and cardiomyocytes and discuss the key questions remaining.

## Cardiac Fibroblasts

The human heart is composed of 5 major cell types: cardiomyocytes, fibroblasts, endothelial cells, macrophages, and smooth muscle cells.^14^, ^15^, ^16^ Cardiomyocytes contribute roughly 30% to 40% by number and roughly 65% to 80% by volume in the adult mammalian heart.^16^, ^17^, ^18^, ^19^, ^20^, ^21^ Recent single‐cell sequencing studies highlight considerable heterogeneity between the different regions of the human heart, with ventricles having distinct cellular signatures compared with atria (47.1% cardiomyocytes compared with 31.1%, respectively).^16^, ^20^ Reports on proportion of cFbs in heart differ, from circa 20% to 60% in rats and humans.^6^, ^15^, ^16^, ^19^ cFb numbers increase with development, aging, and disease. Banerjee et al showed that, in both rats and mice, the number of fibroblasts increases postnatally until adulthood is reached (≈30% to ≈64% in rat and ≈10% to ≈25% in mouse).^15^ An increase in cFbs has also been demonstrated in heart failure and following MI.^14^, ^22^, ^23^

Comparison of transcriptomes has revealed more similarities between cFbs and cardiomyocytes than between other fibroblast cell types.^24^, ^25^ In addition, use of single‐cell RNA sequencing has identified several subpopulations of cFbs within the heart, displaying distinct genetic signatures.^16^ As mesenchymal cells present in every tissue in our body, these subpopulations may be indicative of the various possible cFb lineages.^6^, ^16^ In the heart, cFbs are primarily derived from the proepicardium, but can also arise by epithelial‐to‐mesenchymal and endothelial‐to‐mesenchymal transition.^6^, ^26^ Because of this heterogeneity and their ubiquitous nature, markers specific to cFbs are still lacking. Vimentin is often used alongside specific morphological properties of cFbs, such as the lack of a basement membrane. Some reports have used FSP1 (fibroblast‐specific protein 1) and discoidin domain receptor to identify cFbs, but these are also found in leukocytes and fibrocytes, respectively.^6^, ^27^

Regardless of origin, resident cFbs are dispersed throughout the heart as strands and sheets that sit between cardiac muscle fibers acting as a scaffold.^28^, ^29^ Their main function is to maintain the ECM through regulation of matrix metalloproteinases and tissue inhibitors of metalloproteinases, and therefore mediate secretion and degradation of collagen.^30^ During disease or injury (eg, following MI), cFbs undergo a transition from fibroblast to myofibroblast.^6^, ^31^ This activated phenotype is described as having a spindle shape, increased proliferation, expression of α‐smooth muscle actin (α‐SMA), and increased production of collagen.^32^, ^33^ Single‐cell sequencing in mouse hearts further defined these differences between the activated myofibroblast and nonactivated cFb. They found ≈30 genes differentially expressed between the 2 cell types, including periostin and collagens 1 and 3, all known to be associated with the myofibroblast.^34^

The main role of this cell type is wound healing and repair. Following MI, formation of a fibrotic scar, in place of necrotic tissue, prevents rupture of the myocardium.^4^, ^22^ The expression of α‐SMA stress fibers provides these cells with the ability to contract. This causes shrinkage of the fibrotic scar and produces tension, which, in turn, increases the stiffness of the myocardium.^32^, ^35^, ^36^, ^37^ Although initially beneficial for maintenance of cardiac function and prevention of heart rupture, long‐term effects of excessive fibrosis are detrimental (see section on Cross Talk Between the Myofibroblast and the Cardiomyocyte below).

**Figure 1:**
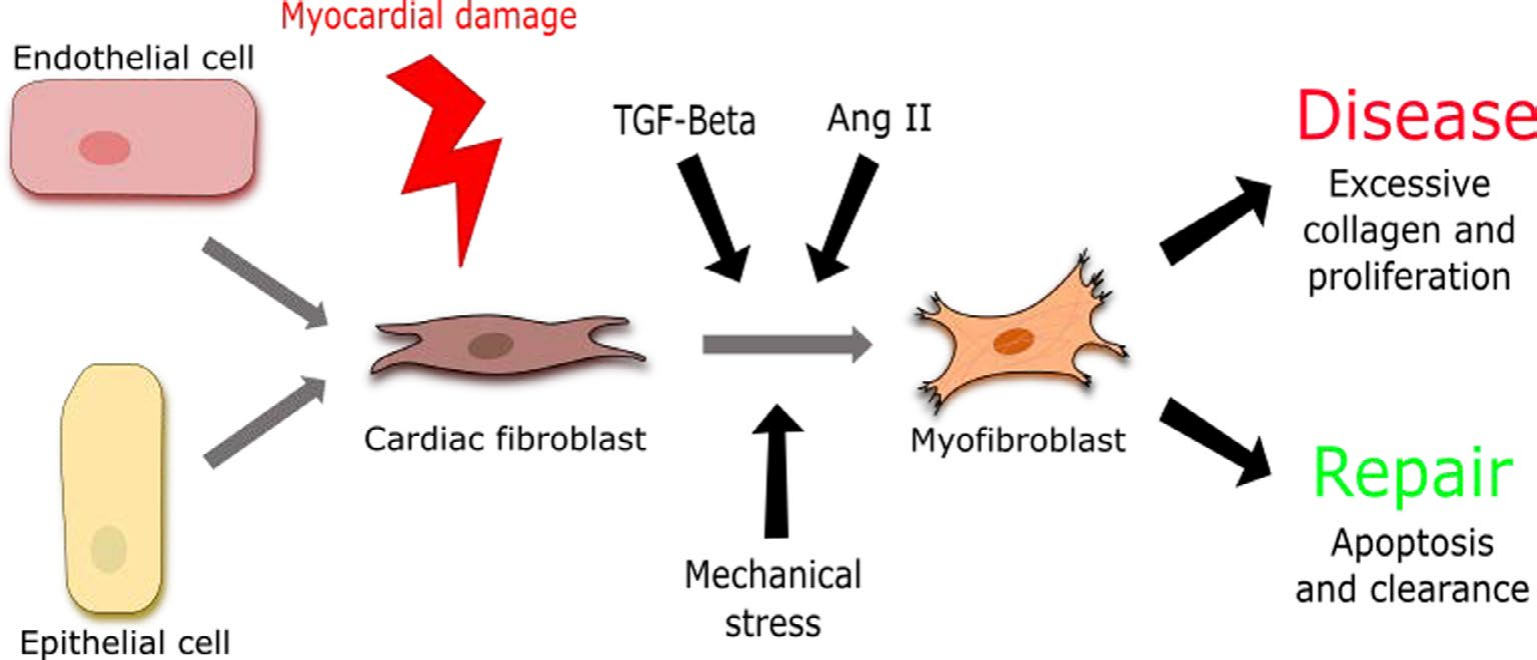
Resident cardiac fibroblasts (cFbs) derive from endothelial‐to‐mesenchymal or epithelial‐to‐mesenchymal transition. Injury to the myocardium initiates signaling pathways that trigger the activation of cFbs to myofibroblasts (MyoFbs). Loss of structural integrity via cardiomyocyte (CM) death also creates mechanical stress that mediates cFb to MyoFb activation. Consequences of MyoFb activation vary between repair and disease processes. There are multiple mechanisms for activation, including mechanical stimuli and paracrine factors, such as transforming growth factor‐β (TGF‐β) and angiotensin II (Ang II), from a variety of different sources, such as CMs and the cFbs themselves.^28^, ^43^

Structural and electrical remodeling of the myocardium, attributed to chronic and proinflammatory conditions, such as hypertension, is commonly associated with cardiac fibrosis.^28^, ^38^, ^39^ Inflammation has been demonstrated to lead to cardiac fibrosis via activation of profibrotic signaling cascades via interleukin 6 (IL‐6) (see section on Interleukin 6 below).^40^ Stiffness itself can activate cFbs, leading to transition to myofibroblasts (Figure 1). Myofibroblasts themselves can secrete further profibrotic factors, driving progression to the kind of fibrosis seen following MI.^28^, ^32^, ^41^, ^42^ Alongside increased stiffness, fibrosis has also been observed to slow conduction and disrupt electrical wave propagation. Ultimately, these factors contribute to decreased cardiac function and increased susceptibility to arrhythmias.^43^

The transition from cFb to myofibroblast is a multifactorial process mediated by several factors (Figure 1).^28^, ^37^ Transforming growth factor‐β (TGF‐β) has been shown in several independent studies to be directly involved in this transition process.^44^, ^45^, ^46^ Myofibroblasts, once activated, can themselves secrete TGF‐β, thus creating a detrimental positive feedback loop leading to sustained fibrosis.^47^, ^48^, ^49^ The importance of tensile strength and mechanical stress in the activation of myofibroblasts has also been demonstrated (see section on Cross Talk Between the Myofibroblast and the Cardiomyocyte for more detail).^32^, ^48^, ^50^ Perhaps not surprisingly, cFb activation can modulate cardiomyocyte function, and several modes of cFb‐cardiomyocyte communication have been uncovered.

## Mechanisms of cFb‐Cardiomyocyte Communication

**Figure 2:**
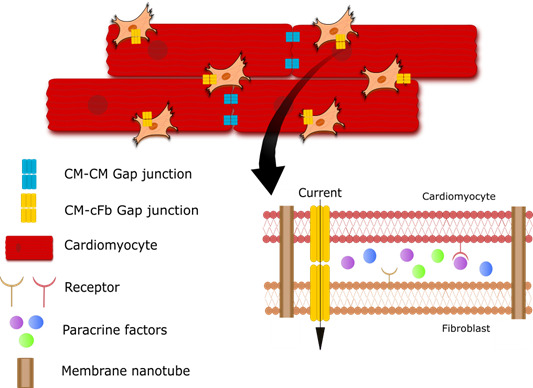
Figure depicting cardiomyocyte (CM)–myofibroblast (MyoFb) communcations. Gap junctions are present at distal junctions between CMs as well as elsewhere between CMs and MyoFbs. Gap junctions and membrane nanotubes allow exchange of molecules between the cytoplasms of cells. Paracrine factors secreted by cells bind receptors on neighbouring membranes, initiating signaling pathways. cFb indicates cardiac fibroblast.

In the 1980s, multiple studies demonstrated cell‐cell interactions between excitatory and nonexcitatory cells in the heart. These were shown to be mediated via paracrine signaling and/or direct contacts between the cells.^51^, ^52^ From the mid‐2000s, there has been more focus on the effects of the nonexcitatory stromal cells on the electrophysiological features of the heart, with changes to conduction velocity and membrane depolarization observed during coculture.^53^ This is perhaps not surprising considering that they make up a significant proportion of the total cell count.^54^ More recently, multiple studies have identified that electrotonic communication occurs between cFbs and cardiomyocytes. This was demonstrated using cultured as well as freshly isolated cFbs in vitro, and in animal disease models in vivo.^13^, ^53^, ^55^, ^56^ The interaction and communication between cardiomyocytes and cFbs was shown to occur through several different modes (Figure 2). These include heterologous coupling through the formation of gap junctions or membrane nanotubes, and via mechanical forces and paracrine signaling.^13^, ^53^, ^57^, ^58^ However, the question still remains as to whether these coupling events occur in vivo in humans.

## Communication via Gap Junctions

Gap junctions are considered the main mechanism for electrotonic coupling in the heart.^59^ They are ion channels that allow propagation of electrical signals between neighboring cells. Gap junctions are formed through the combination of 2 hemichannels, which are either homomeric or heteromeric. Depending on the composition of the 2 hemichannels, the junction will be either homotypic or heterotypic.^60^ The combination of different connexins has demonstrated varying levels of channel permeability.^61^, ^62^ Connexin 40, connexin 43 (Cx43), and connexin 45 are the 3 main connexin subtypes found in the heart. Cx43 is the most widely expressed across the atrial and ventricular myocardium in both cardiomyocytes and cFbs.^59^, ^60^

Changes in the expression of Cx43, and in some cases connexin 45, have been observed to affect cardiac function. Studies have shown that the aging heart is accompanied by abnormalities in expression and distribution of Cx43.^63^, ^64^ When 10‐week‐old rat hearts were compared with those from 2‐year‐old rats, a decreased intensity of Cx43 expression was seen in the left ventricles.^64^ Similar results were also seen in the atria of guinea pig hearts, accompanied by deterioration of adhesive junctions as well as gap junctions.^63^ Furthermore, this age‐related decrease in Cx43 has been linked to slowed conduction velocity.^65^ Together, these results show that through mediating expression of Cx43, aging can lead to conduction slowing and increased risk of arrhythmias.

These changes have also been observed following cardiac injury. Vasquez et al^13^ revealed that Cx43 and connexin 45 are present at cardiomyocyte‐fibroblast contact points in neonatal rat cells. In a model of cardiac injury, an increase in expression of Cx43 in cFbs, along with an observed increase in functional coupling between cardiomyocytes and cFbs, was detected. The same study also identified that myofibroblasts isolated from infarcted hearts, when cocultured with cardiomyocytes, decreased conduction velocities of the action potential and reduced action potential duration at 70% repolarization. More important, the authors showed that the effects on conduction and action potential duration were cFb density dependent, thus pointing toward a potential role in driving electrophysiological heterogeneity across the heart and thus contributing to increase in arrhythmias.^13^ This cFb density dependency on cardiomyocyte electrophysiological features was also shown in modeling studies by Sanchez et al, with effects being most prominent in the left atrial pulmonary vein myocardium, a common site of atrial ectopy.^66^

Zhang et al demonstrated in a mouse model that cFbs are activated following MI. Interestingly, these observations were apparent even in remote regions, away from the site of injury.^67^ This implicates myofibroblasts in the postinfarct remodeling process, as established by other groups.^67^, ^68^ More important, Zhang et al demonstrated that cFbs from infarcted hearts displayed increased expression of Cx43 and intercellular cardiomyocyte coupling. However, in contrast, cardiomyocytes from these models exhibited downregulation and redistribution of Cx43 following MI. Decreased expression of Cx43 in cardiomyocytes has been shown to lead to increased propensity for abnormal conduction and susceptibility to arrhythmias.^67^, ^69^ These data together suggest that cFbs may be maintaining electrical coupling, to some extent, to preserve cardiac function.^67^ Whether this adaptive process is ultimately detrimental to the heart requires further confirmation. With the availability of human induced pluripotent stem cell–derived cFbs and cardiomyocytes, there is an opportunity, and a clear need, for further studies robustly examining the interaction and effects of nonactivated and activated cFbs on cardiomyocyte electrophysiological features in human cell lines.^70^

## Communication via Membrane Nanotubes

Another mechanism that facilitates cFb‐cardiomyocyte communication is that of membrane nanotubes. This was established as a novel interaction by He et al, who demonstrated that membrane nanotubes, through the exchange of Ca^2+^, could facilitate both long‐ and short‐range connectivity between cardiomyocytes and cFbs and therefore regulate cardiac contractility.^57^ Membrane nanotubes are long, thin, membrane‐bound connections that consist of F‐actin and, in some cells, microtubules.^71^, ^72^ They are suggested to help to control structural connectivity between cardiomyocytes and cFbs but also allow direct communication via the exchange of organelles, vesicles, and ions, such as Ca^2+^, across distances of up to several micrometers. In theory, membrane nanotubes have the advantage over gap junctions, which are limited to transfer of molecules of <1.2 kDa and only where cells are in close proximity.^57^ Although membrane nanotubes have been identified in both cardiomyocytes and cFbs in vivo,^57^, ^72^ the proposed effects on contractility have not, and more research is required to identify their role and contributions to mediating electrical signals in the heart.

## Communication via Paracrine Signaling

Cardiomyocytes and cFbs can also interact indirectly via paracrine signaling.^73^ Considering that fibrosis is also demonstrated in areas remote to the injured myocardium, it is perhaps not surprising that paracrine factors may be involved in this process.^74^ To further investigate this concept, several groups have used separated coculture methods, using physical inserts to separate the 2 cell types within the same well or cFb conditioned medium harvested from cultured cFbs.^13^, ^75^, ^76^ These have demonstrated the involvement of paracrine mediators independently and alongside that of mechanical stress or physical coupling on electrophysiological activity.^13^, ^75^ LaFramboise et al showed that, following the addition of cFb conditioned medium, cardiomyocyte hypertrophy as well as reduction of spontaneous contractions were observed in neonatal cardiomyocytes.^75^ Vasquez et al^13^ built on this work by comparing effects of conditioned media obtained from normal hearts and hearts with MI. They showed that conditioned medium from cFbs isolated from rat hearts with MI leads to slowed conduction velocities and a shortened action potential duration when compared with normal cFbs.^13^

There is growing evidence in support of cFb‐produced paracrine factors playing a role in modulation of cardiac function and arrhythmogenesis. Many of these paracrine factors involved are known, however, mechanisms driving the observed changes in cardiac function and are under investigation. These will be discussed in the next section.

## Cross Talk Between the Myofibroblast and the Cardiomyocyte

Following identification of such communications between cFbs‐cardiomyocytes, the importance of defining the consequences of these mechanisms became apparent. The primary focus to date has been on TGF‐β. An important mediator in the differentiation of cFb to myofibroblast, it has also been shown to have direct effects on cardiomyocyte function.^47^, ^72^, ^77^ However, there is also evidence for the involvement of angiotensin II (Ang II), IL‐6, and mechanical stimuli in the communication processes.^72^, ^73^

## Role of TGF‐β

**Figure 3:**
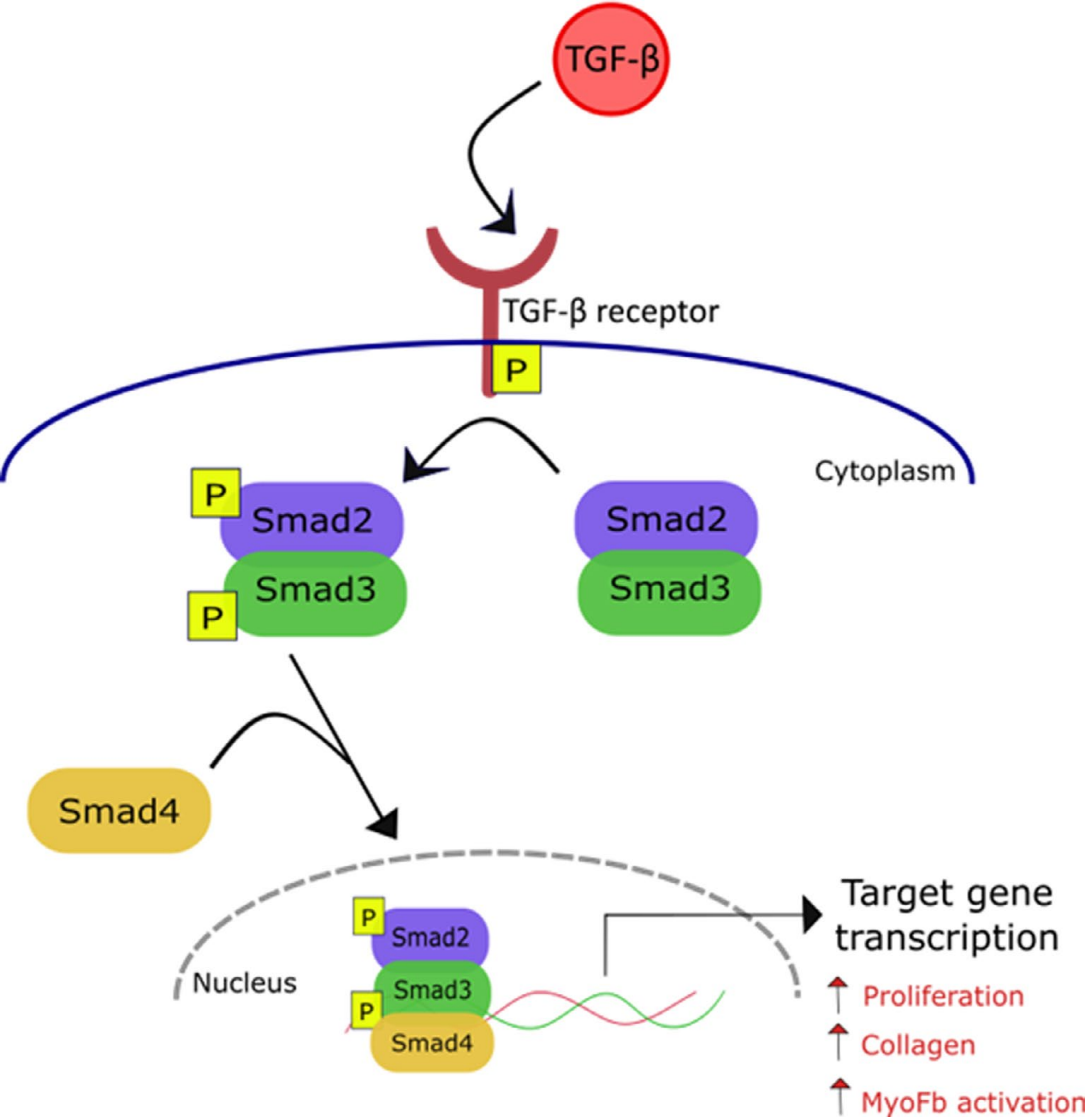
Schematic of transforming growth factor‐β (TGF‐β) activation via small mothers against decapentaplegic (Smad) signaling. TGF‐β binds its receptor, initiating phosphorylation (p) of Smad2/3. This complex then binds Smad4 and translocates to the nucleus to induce transcription of target genes involved in proliferation, collagen production, and activation of cardiac fibroblasts to myofibroblasts (MyoFbs).^42^, ^75^

**Figure 4:**
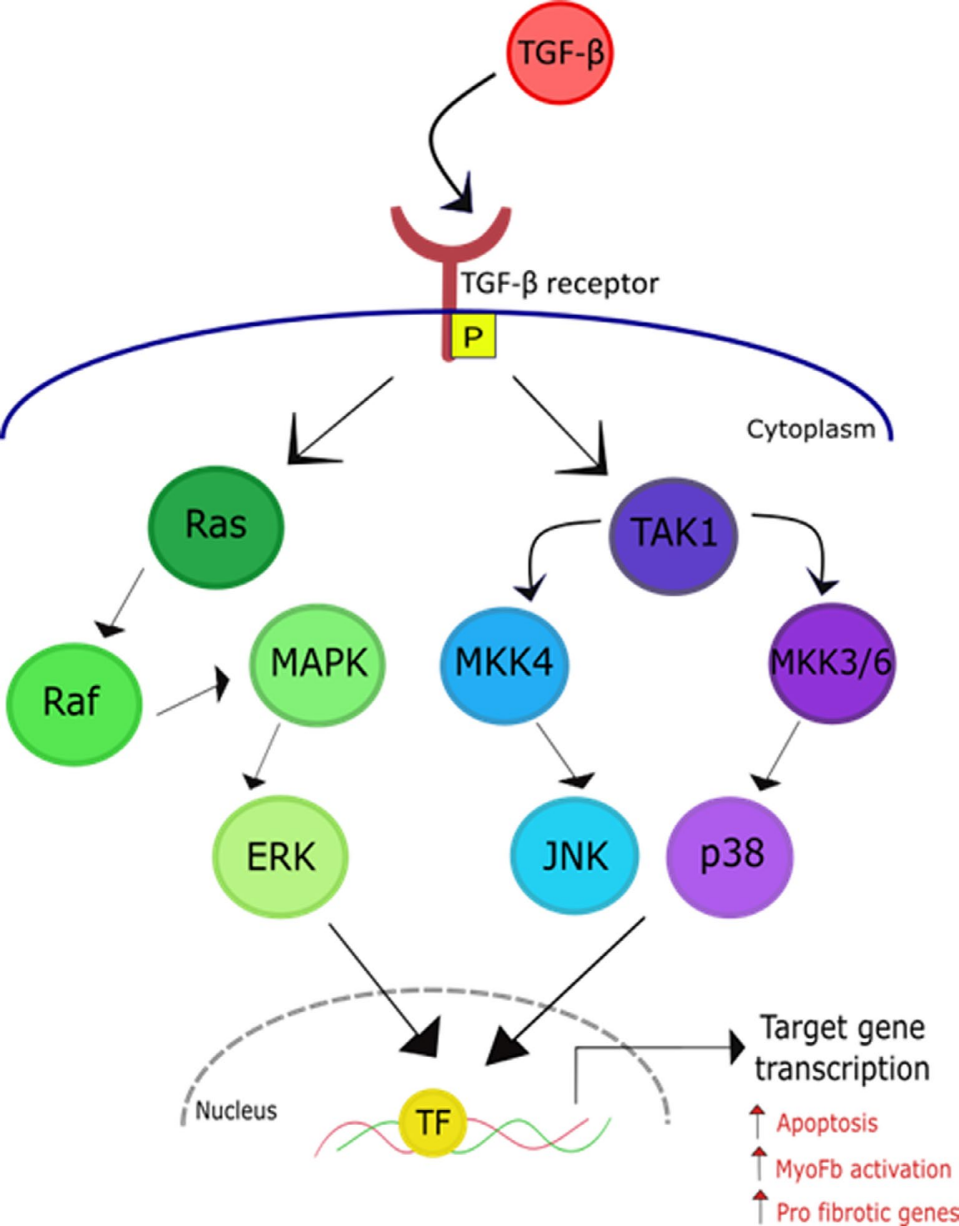
Schematic of transforming growth factor‐β (TGF‐β) activation via noncanonical (small mothers against decapentaplegic–independent) signaling. TGF‐β binds its receptor, initiating activation of Ras or TAK1 and further downsteam activation of extracellular signal‐regulated kinase (ERK), JNK, or p38. This leads to promotion of transcription factors (TFs) and the transcription of target genes involved in myofibroblast (MyoFb) activation, fibrosis, and cardiomyocyte apoptosis.^79^ MAPK indicates mitogen‐activated protein kinase.

TGF‐β is an important cytokine involved in many different cellular processes, including proliferation, differentiation, and migration.^77^ Its expression is altered by several stimuli, such as mechanical stretch, hormones, and cytokines. In the heart, it is the driving force for myofibroblast activation and is therefore crucial during injury and wound healing.^44^, ^78^ TGF‐β receptor stimulation activates multiple downstream pathways, via both small mothers against decapentaplegic (Smad)– (Figure 3) and non–Smad‐mediated transcription (Figure 4), to induce cFb proliferation, collagen synthesis, and myofibroblast activation.^4^, ^47^, ^79^ Multiple studies have shown that TGF‐β activates cFbs, at least to some extent, demonstrated by the expression of α‐SMA fibers and a contractile phenotype.^4^, ^25^, ^47^

In 1988, Thompson et al reported the importance of TGF‐β in cardiac disease in rats. They showed a marked increase in TGF‐β in cardiomyocytes following MI with increased localization to the border zone.^80^ Several studies since then have reported TGF‐β contributing to both fibrotic and hypertrophic effects in the myocardium, both in vitro and in vivo.^4^, ^73^, ^77^ Furthermore, higher levels of TGF‐β are detected in hypertrophic hearts and in heart failure models.^22^, ^81^ These effects have been identified in direct coculture with cFbs and myofibroblasts and through the use of cFb/myofibroblast conditioned medium. In the presence of a TGF‐β receptor inhibitor, these effects were reduced or even reversed. These studies clearly highlight the role of TGF‐β in profibrotic and prohypertrophic processes.^73^, ^82^

Nakajima et al also observed the effects of TGF‐β to be more prominent in the atria compared with the ventricles. Through use of models with constitutive TGF‐β activity, they concluded that TGF‐β alone was not sufficient in promoting ventricular fibrosis in their mouse model.^77^ Rahmutula et al later built on this work using a similar model. They observed an increased expression in TGF‐β signaling proteins in both atria and ventricles, although expression of fibrotic genes was only increased in the atria. This expression was associated with an increased atrial susceptibility to fibrosis and arrhythmia. Similar observations were made in human atrial tissue.^83^ Interestingly, it has also been demonstrated that TGF‐β maintains a positive feedback loop by acting on cardiomyocytes to sustain its own expression and maintain myofibroblast proliferation.^73^, ^84^ Competitive inhibition of TGF‐β receptors can prevent the cFb activation process.^22^ Several independent studies have explored targeting TGF‐β signaling as a potential antifibrotic therapeutic strategy. Kuwahara et al observed, in pressure overloaded mouse hearts, that fibrosis could be decreased using an anti–TGF‐β neutralizing antibody.^49^ Similarly, Khalil et al demonstrated that deletion of *Smad2/3* attenuated the fibrotic response in pressure overloaded mouse hearts. However, increased mortality in TGF‐β receptor knockdown models and following long‐term pharmacological inhibition after MI limits its therapeutic use.^22^, ^44^

Despite this, a study by Davies et al further implicated non‐Smad signaling in atrial remodeling and arrhythmogenesis, specifically with age. Through use of a conditional mouse knockdown of *Mkk4*, they demonstrated MKK4 as a negative regulator of TGF‐β signaling through activation of the JNK pathway. They showed that, with age and with *Mkk4* deletion, there were significantly greater levels of fibrotic tissue, TGF‐β1 expression, TGF‐β receptor expression, and effects on matrix metalloproteinase and tissue inhibitor of metalloproteinase expression levels, providing a substrate for arrhythmogenesis. Furthermore, they discovered that, in patients with atrial fibrillation, there were decreased levels of MKK4.^85^

More recently, TGF‐β has also been linked to the WNT signaling pathway in the context of fibrosis and cardiac tissue remodeling.^86^ Several groups have observed a role for WNT proteins in the activation of myofibroblasts. Blyszczuk et al demonstrated this through the suppression of TGF‐β–mediated myofibroblast activation by blocking WNT signaling.^87^ Others have observed stimulation of proliferation and upregulation of profibrotic genes through activation of the WNT pathway.^86^, ^88^, ^89^ These data, together with those of Davies et al,^85^ suggest that although TGF‐β itself may not be an appropriate target for pharmacological intervention, deciphering downstream targets could in fact provide new avenues for treatment.

The influence of TGF‐β on ionic handling in the heart is less well defined. In coculture with cardiomyocytes, both cFbs (defined as α‐SMA–negative Fbs) and myofibroblasts have been reported to alter Ca^2+^ transients. Myofibroblasts decreased Ca^2+^ transient amplitudes, whereas surprisingly, cFbs increased them. This effect was blocked by a TGF‐β receptor inhibitor. TGF‐β treatment of cardiomyocytes alone was not able to recapitulate the effects seen in coculture systems, indicating that TGF‐β itself does not drive these Ca^2+^ transient alterations.^73^ Kaur et al observed a peak Na^+^ current increase of ≈40% following exposure to TGF‐β. TGF‐β effects on the current magnitude were voltage independent, and these effects were reversed in presence of a TGF‐β neutralizing antibody.^76^ Whether these effects alter action potential morphological features and conduction or whether TGF‐β leads to changes in intracellular Na^+^ concentrations is unclear.

The effects of TGF‐β on cardiac pathogenesis have been recognized for many years through promotion of fibrosis and cardiomyocyte hypertrophy.^49^, ^81^, ^85^ The precise role of TGF‐β in cardiac disease is only now becoming more evident. Numerous studies and the modern ‐omics approaches have highlighted TGF‐β involvement in the modulation of multiple signaling pathways, important in activating the myofibroblast.^44^, ^90^, ^91^ Novel use of computer modeling has enabled generation of in silico fibrosis models,^92^ allowing dissection of the TGF‐β signalosome and potentially identification of novel downstream targets for antifibrotic therapy.

## Angiotensin II

Ang II is a hormone that acts as part of the renin‐angiotensin system and is involved in regulating blood pressure and sodium homeostasis.^93^, ^94^ Inhibition of Ang II signaling through use of angiotensin‐converting enzyme inhibitors has long been used to treat hypertension.^95^ However, evidence over the years has also suggested an important role for Ang II in ventricular remodeling following cardiac injury.^96^, ^97^ Schieffer et al showed that, by blocking angiotensin receptors and by angiotensin‐converting enzyme inhibition, ventricular remodeling was attenuated through reduction of cardiac hypertrophy and fibrosis.^96^

Recent evidence implicates Ang II as a powerful profibrotic factor, as secreted by cFbs/myofibroblasts.^98^, ^99^ Indeed, several independent studies demonstrate that exposure to Ang II stimulates cFb proliferation and, ultimately, fibrotic processes.^97^, ^99^, ^100^ Cao et al observed that Ang II significantly upregulated α‐SMA in neonatal rat cFbs and that this correlated with an initial increase in Cx43 expression, indicative of myofibroblast activation.^90^ In the heart, myofibroblasts have been identified as a significant source of Ang II, implying the existence of a positive‐feedback mechanism.^100^ Ang II also stimulates the release of TGF‐β and IL‐6 from cFbs, which has been shown to further escalate the fibrotic process and the hypertrophic response seen in cardiomyocytes.^99^, ^101^ In patients with hypertensive heart disease, the angiotensin‐converting enzyme inhibitor, lisinopril, reduced myocardial fibrosis, irrespective of its effects on left ventricular hypertrophy.^102^ These clinical findings validate some of the preclinical evidence in favor of Ang II being a major driver of cardiac fibrosis.

## Interleukin 6

IL‐6 is a cytokine involved in processes of differentiation, growth, and survival.^103^ Studies show that the IL‐6 signaling pathway is crucial to cardiac function and cardioprotection following acute injury. However, continuous activation has been associated with attenuated cardiac function attributable to processes including cardiomyocyte hypertrophy and fibrosis.^104^, ^105^ IL‐6 is secreted by both cardiomyocytes and cFbs, with increased expression seen in myocardial hypertrophy.^106^, ^107^ Although cardiomyocytes and cFbs lack IL‐6 receptors, local IL‐6 secretion is likely to promote recruitment of the inflammatory cells to myocardium. Indeed, IL‐6 has clearly been demonstrated to play a prominent role in inflammation.^40^, ^103^

A study conducted by Ma et al used a macrophage/cFb coculture model to observe the effects of Ang II and IL‐6 on the activation of cFbs. They observed that cFbs were the main source of IL‐6 following stimulation of Ang II and that this was reliant on the presence of macrophages. It was also determined that the presence of macrophages stimulated α‐SMA expression and collagen synthesis, and therefore activation of myofibroblasts, in an IL‐6–dependent manner.^108^ These results dictate in part a role for IL‐6 in cardiac fibrosis through inflammatory processes. However, it was subsequently demonstrated that following cardiac injury, cardiomyocytes are stimulated to produce IL‐6, in turn thus stimulating proliferation of cFbs and further IL‐6 secretion. This feedback loop fuels cardiac fibrosis and hypertrophy, leading to decreased cardiac function.^99^, ^106^, ^107^ Furthermore, Meléndez et al demonstrated that elevations in IL‐6 resulted in cardiac fibrosis and a large degree of ventricular stiffness.^40^

The role of IL‐6 has also been investigated using complete genetic knockouts. These mice displayed significant differences when compared with wild type. The hearts were larger while also having thinner chamber walls. Echocardiographic analysis showed cardiac dilation, implying decreased cardiac function. Increased collagen deposition was also observed in knockout mice, along with a higher percentage of cFbs. However, interestingly, abrogation of IL‐6 led to decreased cellular interactions between cardiomyocytes‐cFbs because of a loss of cellular adhesion.^104^ Remarkably, a more recent study, performed by McArthur et al, observed an upregulation of Cx43 expression following stimulation with IL‐6. Although this was only significant when in combination with the soluble IL‐6 receptor, it does point to a greater involvement in these cellular interactions.^109^ This evidence supports the involvement of inflammation, and specifically IL‐6, in myocardial disorder through cardiomyocyte hypertrophy, fibrosis, and a loss of cardiomyocyte‐cFb interactions. However, mechanisms driving the sustained IL‐6 response that leads to these pathogenic transitions are unclear and require further investigation.

## Role of Mechanical Stimuli

The Young modulus is a mechanical property that describes the elasticity of a material.^110^ The healthy myocardium has a Young modulus ranging from 10 to 30 kPa.^48^, ^111^, ^112^ However, following disease or injury, the development of a collagenous fibrotic scar increases the Young modulus to ≥100 kPa.^35^, ^113^ The protein α‐SMA is associated with an increase in contractile force, which is needed for wound contraction following collagen deposition and is therefore used as an activation marker for myofibroblasts.^41^, ^42^

It is now commonly accepted that, under standard tissue culture conditions, because of the stiffer plastic surfaces (≈3 GPa), cFbs undergo activation to myofibroblasts. Several independent studies demonstrated increased expression of α‐SMA after only a few hours of culture in rat cFbs.^13^, ^41^, ^112^ Further evidence for the role of mechanical signals in the cFb‐to‐myofibroblast activation process has been demonstrated through the use of multiple substrates of varying Young moduli to mimic healthy and pathological cardiac environments. Use of higher Young moduli substrates (≥100 kPa) leads to increased proliferation of cFbs, higher levels of α‐SMA, and increased deposition of collagen.^41^, ^112^ How these mechanical signals drive the conversion to myofibroblast phenotype is unclear.^41^ Despite this, most studies to date use culture conditions with stiffer surfaces, thus presumably working with activated cFbs. Future studies should take into account substrate stiffness when designing experiments using cFbs to better match physiological conditions (see section on Cardiomyocyte and Cardiac Fibroblast In Vitro Culture Models). Some groups have begun to experiment with polyacrylamide‐ or poly(ethylene glycol)‐based hydrogels, and others are using silicone.^48^, ^114^ In addition, with several commercially sourced culture dishes with softer Young moduli available, these experiments are now more achievable.

As well as acting as a stimulus for activation, mechanical forces have also been implicated in communicative processes in a phenomenon known as mechanoelectric feedback. The effect of mechanical forces on cardiomyocyte‐cardiomyocyte communications is relatively well investigated^115^, ^116^; however, the role of mechanical signal transmission between cardiomyocytes and cFbs is yet to be elucidated. Intercalated discs are structures found at the distal ends of cardiomyocytes, mediating the mechanoelectric feedback between cardiomyocytes.^117^, ^118^, ^119^ They consist of the fascia adherens, desmosomes, and gap junctions.^117^ Fascia adherens junctions are constructed from adhesion proteins, known as cadherins, that link the intercalated discs to the actin cytoskeleton within the cardiomyocytes.^118^, ^119^ Mutations in cadherins, as well as plakoglobin and desmoplakin, have significantly affected electrical coupling.

Normal electrical coupling of cardiomyocytes has some dependence on normal mechanical coupling, and thus it is reasonable to assume that the mechanoelectric phenomenon also mediates heterocellular coupling of cFbs and cardiomyocytes.^119^, ^120^ This has been evidenced in part through the application of pulsatile stretch, which induced higher expression of proteins involved in both mechanical and electrical coupling in neonatal rat cardiomyocytes.^116^ Zhuang et al used a custom‐made stretch apparatus to apply 10% pulsatile stretch to cells for either 6 or 72 hours. They observed an increase in Cx43 expression, which corresponded to a relatively rapid increase in conduction velocity. This increase in electrical coupling could contribute to the formation of arrhythmia through dysregulated conduction in heterogeneously contracting cardiac tissue.^116^ Conversely, Thompson et al showed that inhibiting myofibroblast contraction and blocking mechanosensitive channels in monolayers of cocultured myofibroblasts and neonatal rat ventricular cardiomyocytes lead to a minimal increase in myofibroblast membraneous expression of Cx43, a significant increase in cadherin expression, and an increase in conduction velocity.^84^ It is difficult to reconcile these divergent findings. Pulsatile stretch and reduced cellular tension are both likely to play a role in disease progression; however, the pathophysiological relevance of these studies remains to be confirmed.

Multiple studies have also shown that growing cFbs or cardiomyocytes on stiffer substrates causes an increase in expression of proteins mediating cell‐cell contact.^84^, ^119^ N‐cadherin is most commonly found between myofibroblasts and cardiomyocytes. By applying pulsatile stretch to cultures of cardiomyocytes and/or myofibroblasts, there was an upregulation in expression of adhesion proteins, such as N‐cadherin, in both cardiomyocytes and myofibroblasts.^116^, ^119^ Thompson et al have also demonstrated that reduced expression of N‐cadherin, via short‐interfering RNA, reversed conduction slowing by myofibroblasts.^121^

Multiple lines of evidence confirm that environmental stiffness affects structural and functional properties of the myocardium. Increased mechanical forces can lead to altered cardiomyocyte electrophysiological features and pathological fibrosis.^32^, ^112^ Better understanding of the mechanisms and key players driving cardiac fibrosis is necessary if we are to develop targeted therapeutic agents.

## Cardiomyocyte and cFb in Vitro Culture Models

Current in vitro cell culture models do not recapitulate the stiffness characteristics of healthy or diseased myocardium, nor do they regularly consider multiple cells types that exist in the heart and their interplay. Considering that standard plastic dishes, because of high tensile strength, can lead to fibroblast activation, some previous work may require validation.^13^, ^72^ Indeed, activation state of the cFb can significantly modulate structural and functional myocardial properties.^13^, ^73^ cFbs and myofibroblasts (TGF‐β–treated or α‐SMA–positive cells) have differential effects on electrophysiological features in cardiomyocytes. More important, in coculture with cardiomyocytes, myofibroblasts often cause a slowing of conduction.^73^, ^84^, ^121^ However, defining what is the tensile strength of healthy human myocardium is not straightforward. Tensile strengths in the range of 5 to 50 kPa have been reported.^36^, ^41^, ^111^, ^112^ Determination of the Young modulus in healthy and diseased human myocardial tissue can be compromised by handling after surgery but equally by different stages of disease. Furthermore, whether the Young modulus varies between the different chambers of the heart is yet to be investigated and should be considered moving forward.

Most experiments are conducted using murine cardiomyocytes and cFbs, despite significant differences in electrophysiological features when compared with humans, which have been reviewed extensively elsewhere.^122^, ^123^ These species differences are thought to contribute to high failure rates of cardiac drugs in clinical trials.^124^ The use of human induced pluripotent stem cell–derived cardiomyocytes (hiPSC‐CMs) may circumvent these problems to some extent. However, hiPSC‐CMs show an immature phenotype, including spontaneous beating, reduced expression of the potassium channels, and a more depolarized resting membrane potential.^124^ The cardiac differentiation process is also a relatively lengthy one.^125^ Methods aimed at improving differentiation efficiency and hiPSC‐CM yield are becoming routine, whereas many groups have shown tangible improvements in maturity.^126^, ^127^, ^128^ Yang et al treated hiPSC‐CMs for 2 weeks with fatty acids and observed a more mature phenotype. This included larger and less circular cell size and a significant increase in sarcomere length. Also observed were increases in calcium transient amplitude and twitch force.^129^ A slightly more established method is the use of mechanical forces during the differentiation process. Through increased substrate stiffness or application of stretch, several independent studies have demonstrated more mature characteristics.^130^, ^131^, ^132^

Cellular interactions are of course also to be considered when developing a culture model for experimentation. Novel methods, allowing differentiation of human induced pluripotent stem cell–derived cFbs, have been developed, opening up opportunities for much needed human coculture experiments.^133^, ^134^ Furthermore, the roles of other cell types are also beginning to be explored.^20^ Hulsmans et al observed the presence of Cx43 at cell‐cell contacts between cardiomyocytes and macrophages. They also demonstrated that, in the presence of macrophages, cardiomyocytes had a more depolarized resting membrane potential and decreased action potential duration.^135^ Use of endothelial and fibroblast coculture methods has also been reported to aid in induced pluripotent stem cell–derived cardiomyocyte maturation.^136^, ^137^, ^138^, ^139^

Consideration of the effects of ECM on cardiomyocyte and cFb structure and function is also necessary. The varying composition of the ECM can alter its properties, such as stiffness, and consequently exert differential effects on cardiomyocytes and cFbs.^28^, ^35^ It is therefore important to replicate the ECM cellular environment as closely as possible in vitro to create representative culture models. Research groups often use collagen coatings during cell culture as collagen is highly expressed in the myocardium.^112^ However, following injury, it has been demonstrated that the collagen composition of the ECM changes to express more collagen 1 than collagen 3.^42^, ^140^ Equally, ECM protein fibronectin was shown to be required for TGF‐β–induced myofibroblast differentiation.^48^ ECM also has positive effects on cardiomyocyte organization in culture, aiding in cellular alignment, similar to that seen in vivo. This has been demonstrated through use of ridged collagen plates, where cardiomyocytes are allowed to connect to the ECM, forming laminae.^141^

More frequently, hiPSC‐CMs are being used in a 3‐dimensional context to mimic the more complex tissue environment that exists in vivo. This concept of cardiac tissue engineering allows interrogation of both cell‐ECM and cell‐cell interactions, not only between cardiomyocytes but also between other cardiac cell types.^72^, ^142^ A recent study by Lee et al established a 3‐dimensional microtissue, using both cardiomyocytes and cFbs, to develop a novel model of cardiac fibrosis.^143^ Research such as this allows more accurate interrogation of cardiac disease and creates new platforms for discovery of more tailored medicine through use of patient‐derived models.

## Is the Myofibroblast Phenotype Reversible?

Previously, it was thought that the myofibroblast was a terminally differentiated phenotype. The only method of reestablishing tissue homeostasis was that of apoptotic clearance after wound healing. However, more recent studies suggest that dedifferentiation or phenotype reversibility is possible and could hold the answer to identifying new therapeutic targets.^47^

Several pathways have been identified in the cFb‐myofibroblast activation process, with TGF‐β being the main target of interest. Indeed, competitive inhibition of TGF‐β receptors leads to a decrease in expression of α‐SMA fibers, a marker for myofibroblast activation.^47^, ^48^ Other research has shown that softer substrates, around or just below what would be expected of the healthy myocardium, also lead to a decrease in expression of α‐SMA fibers.^37^, ^48^ α‐SMA expression levels in these studies did not return to those of quiescent cFbs, suggesting that there may be an intermediate stage, termed the protomyofibroblast,^42^, ^144^ as observed in a liver fibrosis study.^145^ This cFb plasticity was only demonstrated in freshly isolated cells, so the myofibroblast reversibility in long‐term culture on harder substrates needs to be investigated.^22^, ^32^, ^48^ Interestingly, a study by Nagaraju et al observed that myofibroblasts obtained from patients with heart failure had less α‐SMA expression following treatment with a TGF‐β receptor inhibitor. Whether this was dedifferentiation or simply a loss of α‐SMA fibers is unclear.^22^

Prolonged activation of myofibroblasts leads to sustained fibrotic processes within the myocardium, leading to deterioration of heart function. Fibrosis, although ultimately detrimental, is necessary immediately following the myocardial injury, and myofibroblasts are a crucial part of this process. Inhibition of TGF‐β following myocardial injury has led to increased mortality in a mouse model.^22^ Full genetic knockouts of TGF‐β or certain signaling components (ie, Smad2) have embryonic lethality.^44^, ^146^ Therefore, reversal of the cFb‐to‐myofibroblast transition may not be an appropriate therapeutic approach for myocardial injury, although it may be more useful for treatment of chronic CVD, thus preventing the progression toward heart failure.^22^

## Conclusions

Studies over the past 20 years have started to unveil the complexity of the cell‐cell interactions within the myocardium. In this article, we focus on the role of the cFb and the activated myofibroblast in altering the structure and function of the myocardium. Yet, there are still conflicting views as to the effects of these 2 cell types on cardiac electrophysiological features and whether these phenomena occur in humans. To advance the work further, we will require use of the full range of cellular and animal model systems available, while acknowledging the particular strengths and weaknesses each possesses.

There are difficulties involved with in situ experimentation; there are also limitations involved with culturing cFbs in vitro, in particular their propensity for differentiation/activation to myofibroblasts. In addition, considering the immaturity of hiPSC‐CM models, the importance of developing better, more representative fibrosis models is necessary.

Another issue that cannot be avoided is the immense complexity of the pathogenesis of fibrosis. With multiple different and often intersecting pathways, it will be difficult to identify a single valid target to reduce or revert the process. However, only by integrating existing biological and computational platforms with systems biology approaches will further progress be made. Many of these processes are conserved across organs and, therefore, identifying common mechanisms could have huge clinical benefit for many different diseases involving fibrosis.

## Sources of Funding

This work was supported by the British Heart Foundation grants (PG/17/55/33087, RG/17/15/33106, FS/19/12/34204, and FS/19/16/34169 to Dr Pavlovic; SP/15/9/31605, PG/14/59/31000, RG/14/1/30588, RM/13/30157, and P47352/CRM to Dr Denning; and FS/12/40/29712 to Dr Gehmlich); the Wellcome Trust Grants (109604/Z/15/Z to Dr Pavlovic and 201543/B/16/Z to Dr Gehmlich); Animal Free Research UK (AFR19‐20293 to Drs Pavlovic and Denning); National Centre for the Replacement, Refinement, and Reduction of Animals in Research (CRACK‐IT:35911‐259146 and NC/K000225/1 to Dr Denning and NC/T001747/1 to Dr Gehmlich); Accelerator Award (AA/18/2/34218) to Institute of Cardiovascular Sciences at Birmingham; and Oxford British Heart Foundation Centre of Research Excellence (RE/13/1/30181).

## Disclosures

None.
